# First case report: Late‐onset coronary artery spasm after radiofrequency catheter ablation for atrial fibrillation in a Caucasian patient

**DOI:** 10.1002/ccr3.2977

**Published:** 2020-06-22

**Authors:** Takahiro Tsushima, Mohammed Najeeb Osman, Richard A. Josephson, Sergio G. Thal

**Affiliations:** ^1^ Department of Medicine University Hospitals Cleveland Medical Center Case Western Reserve University Cleveland OH USA; ^2^ Division of Cardiology Harrington Heart & Vascular Institute University Hospitals Cleveland Medical Center Case Western Reserve University Cleveland OH USA

**Keywords:** atrial fibrillation, calcineurin inhibitor, catheter ablation, coronary artery spasm, coronary vasospasm, ganglionated plexus

## Abstract

Most of the coronary vasospasms were found intraprocedurally, and it is very rare to see late‐onset vasospasms that happened a few hours after uncomplicated ablations. The recognition of this rare but potentially life‐threatening complication is important to improve the conventional practice of catheter ablation for patients with drug‐refractory atrial fibrillation.

## Graphical abstract

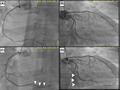

## 1 INTRODUCTION

Radiofrequency catheter ablation (RFCA) is widely performed for patients with drug‐refractory atrial fibrillation. Most of the procedural complications have been significantly reduced over the last decade but RFCA‐induced coronary artery spasm (CAS) has not been fully elucidated. This is the first case report of late‐onset RFCA‐induced CAS in Caucasian patients.

## 2 CASE PRESENTATION

A 57‐year‐old Caucasian man was originally admitted to our cardiac intensive care unit (CICU) for postprocedural observation after his successful radiofrequency catheter ablation (RFCA) for persistent atrial fibrillation (AF). His AF had been refractory to multiple attempts of direct current cardioversion and pharmacological rate control. He has previous history of cirrhosis and concurrent hepatocellular carcinoma due to chronic hepatitis C viral infection and alcoholism. He underwent a successful liver transplantation several years ago, and he is on lifelong immunosuppression with calcineurin inhibitor (tacrolimus). Pharmacological rhythm control was not attempted out of concern for medication interactions with tacrolimus, and he was referred to our hospital for RFCA. He had no previous history of coronary artery disease. He was taking digoxin, metoprolol succinate, warfarin, and tacrolimus. He actively smoked a half pack of cigarettes daily but denied any use of recreational drugs. The preprocedural transthoracic echocardiography (TTE) demonstrated normal left ventricular ejection fraction (60‐65%) without significant valvular diseases, and his warfarin had been continued until the morning of the planned RFCA.

**FIGURE 1:**
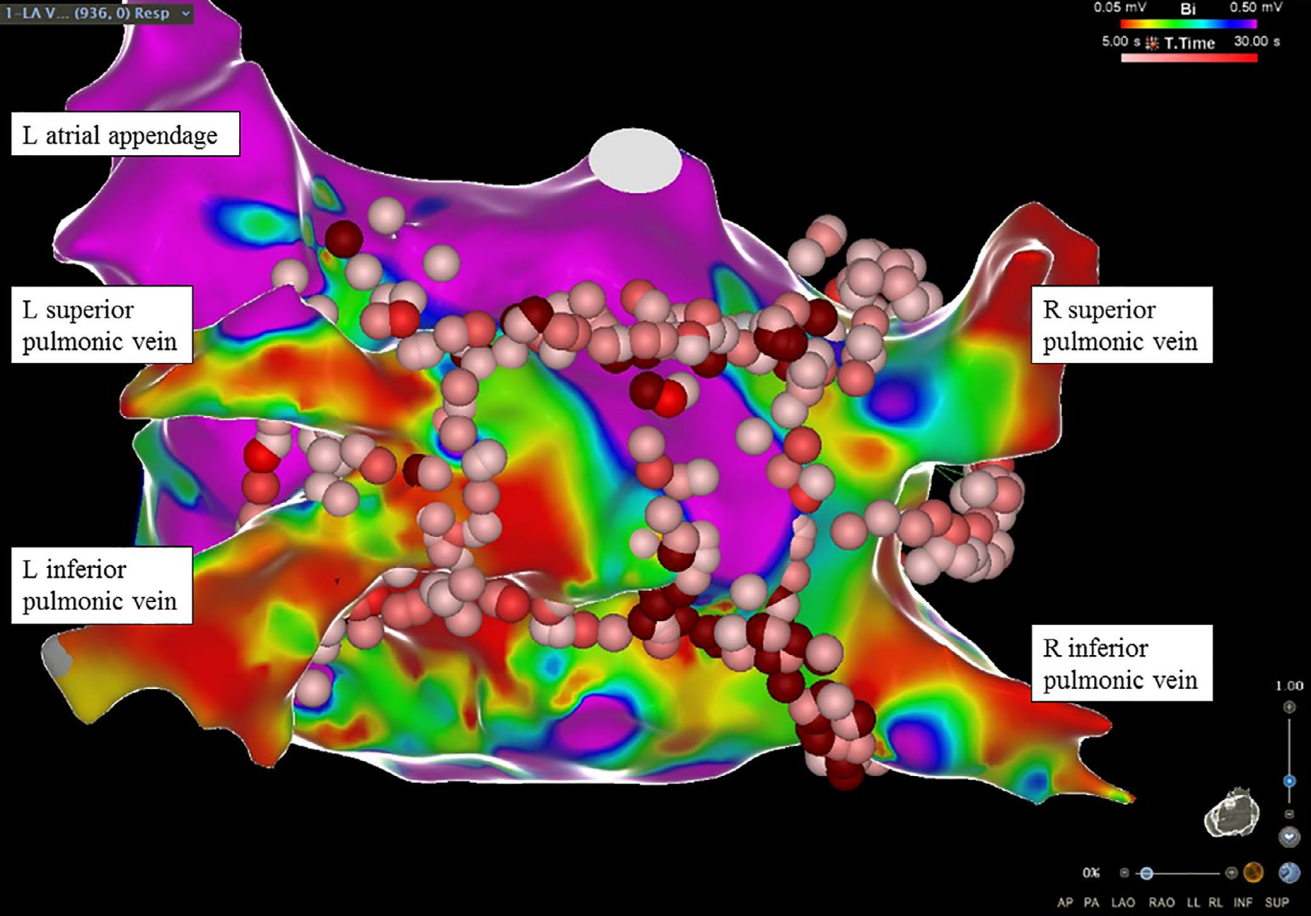
The endocardial electro‐anatomical mapping at the end of the successful radiofrequency catheter ablation in our case (posterosuperior view)

In the electrophysiology laboratory, the left atrium (LA) voltage map was constructed with dragging technique and VISITAG module on the CARTO3 system (Biosense Webster, Inc., Irvine CA). An open irrigated catheter delivered 30 watts continuously without adjusting the power according to different LA sites. A complete isolation of all four pulmonary veins (PV) was achieved with wide‐area circumferential radiofrequency ablation in addition to carinal and posterior box lines (Figure 1). During his RFCA procedure, patient was treated with intravenous heparin infusion with a target activated clotting time of 350‐400 seconds and the intracardiac echocardiography did not demonstrate acute or chronic thrombus since the beginning. Patient was hemodynamically stable without showing acute ST‐T changes on the intraprocedural 12‐lead electrocardiogram (ECG) monitoring. He was successfully converted to normal sinus rhythm and was admitted to CICU for postprocedural observation.

**FIGURE 2:**
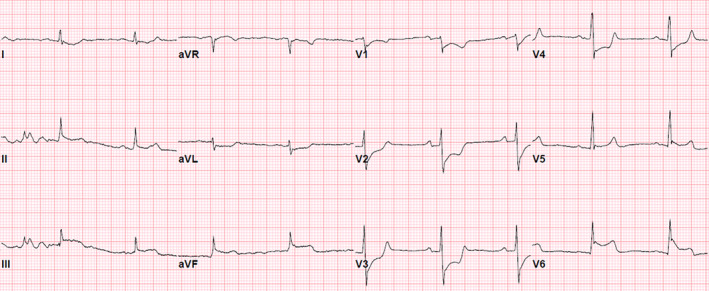
The twelve‐lead electrocardiogram at the onset of initial chest pain

**FIGURE 3:**
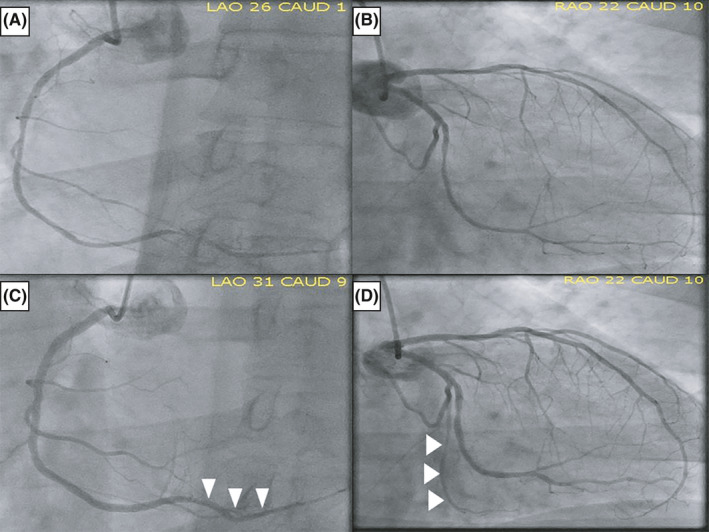
Coronary angiogram before and after injecting intracoronary nitroglycerin. The initial angiogram showed diffuse coronary artery spasm that was more pronounced in the posterolateral branch of the left circumflex artery and the posterior descending branch of the right coronary artery (Figure 3A‐B). These lesions were completely resolved with intracoronary administration of nitroglycerin, and there were no residual obstructions with bubbles or thrombus (white arrow head at Figure 3C‐D)

Three hours after he came to CICU, patient developed progressively worsening chest pain that was described as substernal pressure, 10/10 on subjective intensity, radiating to his left shoulder, and associated with nausea and diaphoresis. He also developed symptomatic hypotension and bradycardia (40‐50 bpm). A repeated ECG demonstrated sinus bradycardia and new ST‐segment elevations in the inferolateral leads (II, III, aVF, V5‐6) with reciprocal ST‐segment depressions in the precordial leads (V1‐4; Figure 2). Therefore, acute ST‐segment elevation myocardial infarction (STEMI) was suspected and he was taken to our cardiac catheterization laboratory for urgent coronary angiography. The initial angiogram showed diffuse spasm of both right and left coronary arteries, and these lesions were completely resolved with intracoronary administration of nitroglycerin (Figure 3A‐D). There were no residual obstructions with bubbles or thrombus, but the spasm was more pronounced in the posterolateral branch of the left circumflex (LCx) artery and the posterior descending branch of the right coronary artery (RCA; white arrow head at Figure 3C‐D). With the administration of intravenous fluids and intracoronary nitroglycerin, his hypotension and chest pain improved quickly with subsequent resolution of the ST elevation in the 12‐lead ECG. The initial troponin I was 3.69 ng/mL (normal range: 0.00‐0.03). Based on these findings, he was diagnosed with acute inferolateral STEMI associated with RFCA‐induced coronary artery spasm (CAS). His home metoprolol and digoxin were held, and he was readmitted to the CICU with continuing intravenous nitroglycerin.

Overnight, the patient developed a different type of substernal chest pain that was pericarditic nature, 5/10 on subjective intensity, and exacerbated with deep inspiration. This was not associated with any hemodynamic instability or ECG changes. The repeated TTE demonstrated preserved left ventricular ejection fraction (50%‐55%), regional hypokinesis at both basal inferolateral and inferior segments, and trivial pericardial effusion. Oral colchicine was initiated for RFCA‐related acute pericarditis. The rest of his hospital course was uncomplicated, and his repeated ECG did not show subsequent abnormal Q wave or ST‐T changes chronologically. The newly started nitroglycerin drip was switched to oral diltiazem, and patient was discharged home with continuing warfarin, tacrolimus, and oral diltiazem 120mg daily. During his outpatient follow‐up, he did not have further episodes of recurrent chest pain or CAS but developed recurrent AF. In addition to the recently initiated diltiazem, the dose‐reduced dofetilide 250 mcg twice daily was newly started and it converted him back to normal sinus rhythm.

## 3 DISCUSSION

Most of the RFCA‐induced CAS were reported to occur during the procedure, unlike our patient who developed CAS three hours after completing the uncomplicated RFCA. There are only five case reports of such a late‐onset CAS after AF ablation in the last decade and all of them were Asian patients (Table 1).^1^, ^2^, ^3^, ^4^, ^5^ Therefore, this is the first case report of late‐onset RFCA‐induced CAS in Caucasian patients.

**TABLE 1:** Available case reports of late‐onset coronary artery spasm after catheter ablation

Reference/Published year	Age/Gender	History of CAS	Onset of CAS after ablation	Diagnosed arrhythmia/Performed procedure	Involved CA	Cardiac complication	Required treatment for resuscitation
1/2009	81/F	ND	8 h	type B WPW syndrome/Right accessory pathway RFCA	Total occlusion of midportion of RCA	VF	Defibrillation, IC nitroglycerin
2/2014	62/M	Yes	1 h	AF and AFL/PVI and CTI block via RFCA	RCA and LCx	cardiac arrest/VF	Defibrillation, IC nitroglycerin
3/2016	73/M	ND	2.5 h	AF and AFL/PVI via Cryoballoon and CTI block	ND	None	IV nitroglycerin
4/2017	68/M	ND	1 h	AF/PVI and SVC isolation via RFCA	ND	None	ND
5/2018	73/M	ND	2 h	AF and AFL/PVI and CTI block via RFCA	ND	AVB and subsequent VF	Defibrillation, IV nitroglycerin

Abbreviations: AF, atrial fibrillation; AFL, atrial flutter; AVB, atrioventricular block; CA, coronary artery; CAS, coronary artery spasm; CTI, cavotricuspid isthmus;F, female; IC, intracoronary; IV, intravenous; LCx, left circumflex; M, male; ND, not documented; PVI, pulmonary vein isolation; RCA, right coronary artery; RFCA, radiofrequency catheter ablation; SVC, superior vena cava; VF, ventricular fibrillation; WPW, Wolff‐Parkinson‐White.

The pathophysiology of RFCA‐induced CAS has not been fully elucidated but there are several suggested mechanisms: pre‐existing endothelial dysfunction of coronary artery, transmural myocardial inflammation from direct thermal damage, medication‐induced vasospasm, and stimulating ganglionated plexus (GP) of LA epicardial adipose tissue (EAT).^6^ In our patient, coronary angiogram demonstrated diffuse CAS profoundly affecting the posterolateral branch of LCx and the posterior descending branch of RCA. These CAS lesions opened widely immediately after the administration of intracoronary nitroglycerin (white arrow head at Figure 3C‐D). Such a diffuse reversible obstruction is not a consequence of air or thrombus embolism or direct thermal injury of myocardium during RFCA. In patients with successful RFCA for right‐side common atrial flutter, the anatomical proximity of the RCA to the ablation site (cavotricuspid isthmus) can cause CAS or thromboembolism at RCA via the direct thermal injury of myocardium.^7^ However, the localized inflammation cannot explain the diffuse CAS involving other major coronary arteries like our patient.

Human histopathologic studies revealed that EAT contained GP regulating cardiac autonomic balance. Endocardial RFCA can hypothetically affect the autonomic activity of GP via direct thermal injury.^8^ It has been identified that there are five major epicardial GP around the LA: left superior and inferior, right superior and inferior, and Marshall vein tract.^8^ Significant vagal reflex and hypotension were observed during these GP ablations and such an enhanced parasympathetic tone (Bezold‐Jarisch reflex) was similar to the initial presentation of our patient.^8^ Our patient developed acute pericarditis later, and this reflects the transmural inflammation spreading to the EAT. Sakamoto et al reported the electro‐anatomical association between the RFCA sites and the intraprocedural ECG changes in detail.^6^ During RFCA of the anterior site of bilateral superior pulmonary veins those were close to the epicardial GP, they observed multiple episodes of transient ST‐segment elevations in the inferior leads. Patients subsequently developed ventricular fibrillation after completing RFCA at the anterior carina of the right pulmonary vein.^6^ Their coronary angiogram revealed multivessel CAS, and these spastic lesions were widely opened after intracoronary bolus of nitroglycerin.^6^ Our RFCA blocking line covered the anterior site of bilateral superior pulmonary veins as well as other epicardial GP (Figure 1).

In patients with paroxysmal AF, Kawakami et al performed CAS provocation with an intracoronary administration of acetylcholine or ergonovine.^9^ CAS was more frequently provoked in patients with paroxysmal AF in comparison with the control group (76.5% vs 8.8%; odds ratio, 33.583; 95% confidence interval, 6.5732‐171.58; *P* < .0001).^9^ All CAS were observed in the proximal portion of right or left coronary artery, and these locations were associated with the distribution of epicardial GPs.^9^ Based on these findings, we believe the multivessel CAS in our patient was mainly provoked by acute autonomic imbalance due to the transmural inflammation of epicardial GP during RFCA.

Besides more, our patient had multiple medical comorbidities contributing to CAS. It is well known that pre‐existing coronary atherosclerosis can lead to CAS owing to coronary endothelial dysfunction and our case has a significant smoking history. Because of multiple medical comorbidities following liver transplantation, liver transplant recipients have a greater risk of cardiovascular death and ischemic events than age‐ and sex‐matched nontransplanted patients.^10^ Also, calcineurin inhibitors (cyclosporine or tacrolimus) are known vasoconstrictors and hypothetically this medication can cause CAS too.^11^ The mechanism of tacrolimus‐induced vasospasm is still unclear but it is suggested that tacrolimus creates an imbalance between the relaxing and contracting factors on the endothelium.^11^

## 4 CONCLUSION

This is the first case report of late‐onset RFCA‐induced CAS in Caucasian patients, and this complication could be provoked by multifactorial pathophysiology, especially acute autonomic imbalance associated with transmural inflammation of epicardial GP during RFCA. Recently, cryoballoon‐induced CAS is also reported.^3^, ^12^ As more extensive and complicated ablations are performed for drug‐refractory AF, the incidence of ablation‐induced CAS may increase. Therefore, the postprocedural observation and recognition of this rare but potentially life‐threatening complication will improve the conventional practice of catheter ablation for patients with drug‐refractory atrial fibrillation.

## CONFLICT OF INTEREST

None declared.

## AUTHOR CONTRIBUTIONS

TT: provided the CICU care and wrote the entire manuscript. MNO: performed the urgent cardiac catheterization and revised this manuscript extensively. RAJ: provided the CICU care and revised this manuscript extensively. SGT: performed the radiofrequency catheter ablation and revised this manuscript extensively.
